# Changes in regional distribution of lung sounds as a function of positive end-expiratory pressure

**DOI:** 10.1186/cc7871

**Published:** 2009-05-10

**Authors:** Shaul Lev, Yael A Glickman, Ilya Kagan, David Dahan, Jonathan Cohen, Milana Grinev, Maury Shapiro, Pierre Singer

**Affiliations:** 1 Department of General Intensive Care, Rabin Medical Center, Beilinson Campus, 39 Jabotinski Street., Petach Tikva, 49100, Israel; 2 Deep Breeze, Ltd., 2 Hailan St., P.O. Box 140, North Industrial Park, Or-Akiva, 30600, Israel

## Abstract

**Introduction:**

Automated mapping of lung sound distribution is a novel area of interest currently investigated in mechanically ventilated, critically ill patients. The objective of the present study was to assess changes in thoracic sound distribution resulting from changes in positive end-expiratory pressure (PEEP). Repeatability of automated lung sound measurements was also evaluated.

**Methods:**

Regional lung sound distribution was assessed in 35 mechanically ventilated patients in the intensive care unit (ICU). A total of 201 vibration response imaging (VRI) measurements were collected at different levels of PEEP between 0 and 15 cmH_2_O. Findings were correlated with tidal volume, oxygen saturation, airway resistance, and dynamic compliance. Eighty-two duplicated readings were performed to evaluate the repeatability of the measurement.

**Results:**

A significant shift in sound distribution from the apical to the diaphragmatic lung areas was recorded when increasing PEEP (paired t-tests, *P *< 0.05). In patients with unilateral lung pathology, this shift was significant in the diseased lung, but not as pronounced in the other lung. No significant difference in lung sound distribution was encountered based on level of ventilator support needed. Decreased lung sound distribution in the base was correlated with lower dynamic compliance. No significant difference was encountered between repeated measurements.

**Conclusions:**

Lung sounds shift towards the diaphragmatic lung areas when PEEP increases. Lung sound measurements are highly repeatable in mechanically ventilated patients with various lung pathologies. Further studies are needed in order to fully appreciate the contribution of PEEP increase to diaphragmatic sound redistribution.

## Introduction

The use of acoustic monitoring technology offers the potential for a radiation-free, noninvasive bedside assessment of lung abnormality in patients during their stay in the intensive care unit (ICU). Correlation between breath sound recordings and regional distribution of pulmonary ventilation has been previously established, particularly in studies conducted by Ploy-Song-Sang and colleagues and other groups who compared acoustic findings with data obtained with radioactive gases [1-3]. The effect of airflow and volume on the amplitude and spectral content of breath sounds has been extensively studied in healthy [4-9] and diseased lungs [10-12]. Furthermore, several studies assessed the effect of changes of mechanical ventilation on lung sound distribution in animal models [13-17]. Räsenen and colleagues reported that the acoustic changes associated with oleic acid-induced lung injury allow monitoring of its severity and distribution [13] and that acute lung injury (ALI) causes regional acoustic transmission abnormalities that are reversed during alveolar recruitment with positive end-expiratory pressure (PEEP) [14]. Recently, Vena and colleagues reported a reduction of amplitude and a change in spectral characteristics of normal lung sounds when increasing PEEP in mechanically ventilated pigs [15]. Finally, recording of crackle-sound during mechanical ventilation was employed to monitor lung recruitment–derecruitment in a porcine model [16,17].

The experience on acoustic monitoring in mechanically ventilated patients is limited [18,19] and only preliminary investigations were conducted to assess changes in regional distribution of lung sound as a function of changes in mechanical ventilator setting [20,21]. Waitman and colleagues classified breath sounds recorded in an intensive care setting using different neural network configurations [22], and a computerized respiratory sound monitor was used to detect wheezes in pediatric ICU [23]. Detection of endobronchial [24-26] and esophageal [27] intubation using lung sound monitoring during anesthesia was also described. Dellinger and colleagues recently reported the use of an acoustic-based imaging device to map the geographical distribution of breath sound as a function of mechanical ventilation mode [28]. Changes in lung sound distribution map during recruitment maneuver and PEEP increase were also reported in four abstracts [29-32]. These findings suggest that breath sound information can be used to evaluate lung condition during mechanical ventilation; however, information regarding lung sound monitoring to adjusted PEEP levels is lacking. PEEP setting is widely used by physicians and respiratory therapists in order to improve gas exchange, mainly in patients with severe hypoxic respiratory failure such as acute respiratory distress syndrome (ARDS) and ALI [33] while preventing end-expiratory alveolar collapse [34] and inspiratory overinflation [35]. In practice PEEP setting is adjusted to patient condition up to several times a day, although no standardized method to adjust PEEP has been accepted to date. The first step in the evaluation of a new approach is to assess if a change in PEEP induces any change in the measurement.

The aim of the present study was to evaluate the effect of changes in PEEP on the regional distribution of lung sounds as recorded by vibration response imaging (VRI), an acoustic monitoring technology that creates a dynamic two-dimensional functional image of lung sound distribution during mechanical ventilation. Repeatability of lung sound measurements was also evaluated.

## Materials and methods

### Patients

The study was performed in the general ICU of the Rabin Medical Center in Petach-Tikva, Israel. The study protocol was approved by the Institutional Review Board and informed consent was obtained from all patients or their next-of-kin. Intra-individual differences in lung sound measurements were investigated at different levels of PEEP in a prospective trial.

### Inclusion and exclusion criteria

Patients enrolled in the study were adults (18 to 85 years old) with a body mass index greater than 21. Exclusion criteria included a body habitus or skin condition that would interfere with sensor placement; the presence of a cardiac pacemaker, implantable defibrillator or artificial heart valve; or pregnancy.

### Study design

The levels of PEEP and the fraction of inspired oxygen (FiO_2_) were adjusted according to clinical requirements. Among the 35 patients enrolled in this study, one was recorded at PEEP 5 and 10 cmH_2_O and 34 at PEEP 0, 5 and 10 cmH_2_O. Fifteen of these 34 patients were also recorded at PEEP 15 cmH_2_O. In 28 patients, PEEP was assigned from low to high level. In order to assess any effect due to the lack of randomization, PEEP levels were applied in a random order in a subgroup of patients (n = 7). At the later stage of the protocol, repeatability was tested on 26 patients for whom two repeated consecutive measurements were performed at the same level of PEEP under the same conditions, over a period of time not exceeding five minutes. No recording was excluded from the repeatability study. Measurements at different PEEP levels were performed at an interval of at least five minutes. No intervention, except for changes in PEEP, was allowed by the protocol. Mode of mechanical ventilation, tidal volume (VT), respiratory rate (RR), partial arterial pressure of oxygen (PaO_2_), FiO_2_, oxygen saturation (SpO_2_), airway resistance (Raw) and online dynamic compliance (Cdyn) as provided by the ventilator were documented. Three consecutive measurements of Cdyn were averaged in order to reduce variability. To keep consistency and ensure that timing between spontaneous and controlled cycles do not affect the results, the spontaneous breath was used whenever available (31 out of 35 patients, 89%), including in synchronized intermittent mandatory ventilation mode (SIMV).

### Recording procedure

**Figure 1:**
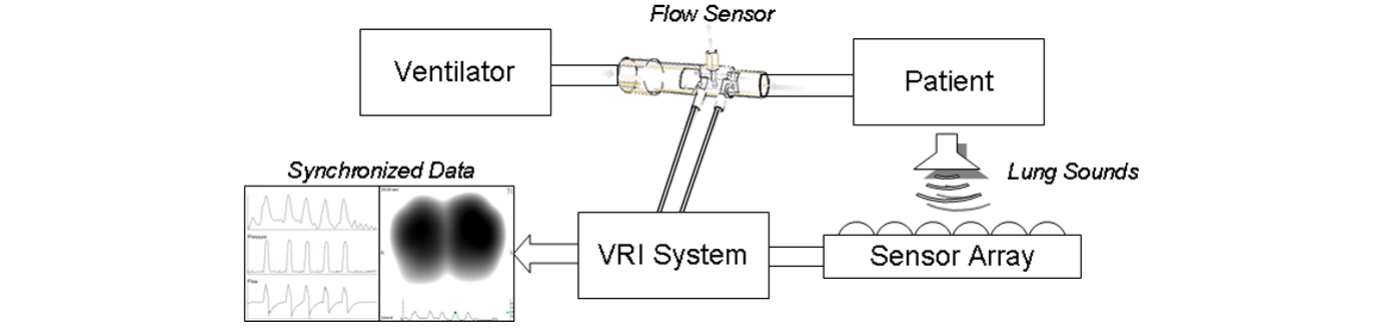
Schematic diagram describing the elements of the system.  The patient lies on the acoustic sensor array and the flow sensor is inserted in the breathing circuit. The vibration response imaging (VRI) system collects acoustic information simultaneously from the sensor array and pressure and flow waveforms from the ventilator.

**Figure 2:**
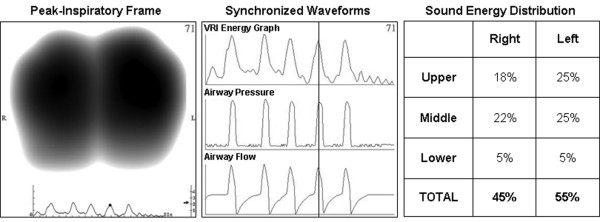
An example of acoustic data as displayed for a recording obtained from a 77-year-old male with myasthenia gravis.  A representative peak-inspiratory image (left panel); synchronized sound energy graph and ventilator airway pressure and flow waveforms (middle panel); sound energy distribution in the six lung regions as automatically provided by the software in percentage of weighted pixel count (right panel). VRI = vibration response imaging.

A schema of the apparatus is provided in Figure 1. The recordings were performed using a VRIxv™ device (Deep Breeze Ltd., Or-Akiva, Israel) with two arrays of six rows by three columns sensors or microphones similar to those used in digital stethoscopes. The recordings were made in supine position with a bed angle between 30 to 45°. The arrays were positioned posterior to the patient's back using a disposable positioning unit to reduce risk of cross-contamination. Morphological landmarks such as spine and scapula were used in order to ensure accurate and repeatable placement of the sensor arrays. Excessive secretions were removed by endotracheal and oral suctioning before each series of recordings. Airway pressure and flow waveforms were sampled from the ventilator using a proximal flow sensor inserted in the patient's circuit. As displayed in Figure 2, these waveforms were synchronized with the sound energy graph representing the average sound energy in both lungs. Each recording lasted for 20 seconds of acquisition time, followed by 40 seconds of processing time, and was stored digitally on the device for later review and analysis.

### Measurement output

The output of the measurement consisted of a normalized dynamic image synchronized with airway pressure and flow waveforms, revealing the geographical distribution of sound energy during the respiratory cycle. Because of image normalization, the measurement output aimed at describing the relative airflow distribution in the lung rather than the absolute volume. As described in detail by Dellinger and colleagues [36], this dynamic image was created from a series of gray-scale still images or frames with each frame representing 0.17 seconds of sound energy. The digitized acoustic signals were band-pass filtered between 150 and 250 Hz to remove heart and muscle sounds; median filtering was applied to suppress impulse noise, and truncation of samples above an automatically determined signal-to-noise threshold was performed. Sound energy was obtained following down-sampling.

Recording quality was assessed according to pre-determined criteria [28]. The graph representing the average sound energy as a function of time throughout the respiratory cycle in both lungs was displayed underneath the dynamic image. Each 20 second measurement included up to 10 respiratory cycles. A normalized representative frame (or map) at peak-inspiratory flow was automatically selected and displayed on the screen (Figure 2). This map was also quantified by the software and presented as the percentage of weighted pixels in six lung regions: upper right (UR); middle right (MR); lower right (LR); upper left (UL); middle left (ML) and lower left (LL), up to a total of 100%. According to the recording procedure, 'lower' corresponded to the diaphragmatic lung region and 'upper' to the apical lung region. The percentage of sound energy in the total right lung (TR = UR + MR + LR) and total left lung (TL = UL + ML + LL) were also documented. The apico-diaphragmatic ratio (ADR), defined as the ratio between the lung sound distribution in the apical lung areas (UR + UL) and the diaphragmatic lung areas (LR + LL) (ADR = (UL + UR)/(LL + LR)), was used to assess changes in lung sound distribution along the cephalocaudal axis. A larger ratio suggested increased sound in the apical areas and a smaller ratio increased sound in the diaphragmatic areas. Distribution was considered more heterogeneous if the difference was larger than two; this threshold was derived from experience with healthy patients in supine positions.

### Statistical analysis

Data are presented as the mean ± standard deviation. When two recordings were performed under identical conditions, the second recording was used for all analysis not related to the repeatability aspect of the study. For samples including more than 30 measurements, paired student's t-test (Microsoft^® ^Office Excel 2003, Microsoft Corporation, Redmond, WA, USA) was used. For the analysis of samples including less than 30 measurements, Wilcoxon matched-pairs signed-ranks test, Friedman two-way analysis on ranks test and Wilcoxon two sample test were used (IFA Services Statistics, Amsterdam, Holland). Friedman test was used to compare three or more paired groups. Coefficients of determination (R^2^) and coefficients of variation (CV) were used to test repeatability. A *P *< 0.05 was considered significant.

## Results

A total of 35 mechanically ventilated patients (26 males, 9 females, age 62 ± 20 years) were enrolled in the study between April 2007 and January 2008. Patients were ventilated using one of two types of ventilators (Puritan Bennett, Tyco Healthcare, Mansfield, MA, USA; Evita XL or Evita 4, Draeger, Lübeck, Germany). The majority of the patients (n = 26; 74%) were mechanically ventilated on pressure support ventilation mode (PSV) with a level between 8 and 24 cmH_2_O (mean 14 ± 4 cmH_2_O). Six patients (17%) were ventilated using SIMV. The rest of the patients (n = 3; 8%) were ventilated with other modes of mechanical ventilation. Patients were not deeply sedated and none were paralyzed.

A total of 201 valid recordings were performed on the 35 patients. No adverse event related to the measurement was registered. Ten recordings (less than 5% of the overall data) were excluded from the analysis based on pre-determined criteria as mentioned above [28]. Poor recording quality was confirmed by an average sound energy level below a pre-defined threshold (< 1 in the energy bar of the imaging display). Reasons for mechanical ventilation of these 35 patients are described in Table 1. Chest radiography results revealed that 19 of these patients had bilateral disease, 13 had unilateral lung pathology inducing decreased lung sounds (i.e. one-lung atelectasis, pneumothorax, or pleural fluid) and three had normal lungs. Average VT was 551 ± 126 mL, SpO_2 _97 ± 3%, RR 21 ± 7 breaths/minute, Cdyn 60 ± 42 mL/mbar, and Raw 16 ± 5 mbar L/second. These parameters did not significantly change with PEEP (Friedman test, paired groups).

**Table 1:** Reason for mechanical ventilation in 35 patients

**Reason for intubation**	**N**	**%**
Pneumonia	8	23
Acute respiratory failure	7	20
Severe chest trauma	3	9
Interstitial lung disease	2	6
Cerebrovascular accident	2	6
Congestive heart failure	2	6
Acute respiratory distress syndrome	2	6
Pancreatitis	2	6
Head trauma	2	6
Others*	5	14

*Sepsis, chronic obstructive pulmonary disease, myasthenia gravis, failure to wean, mesenterial ischemia.

**Figure 3:**
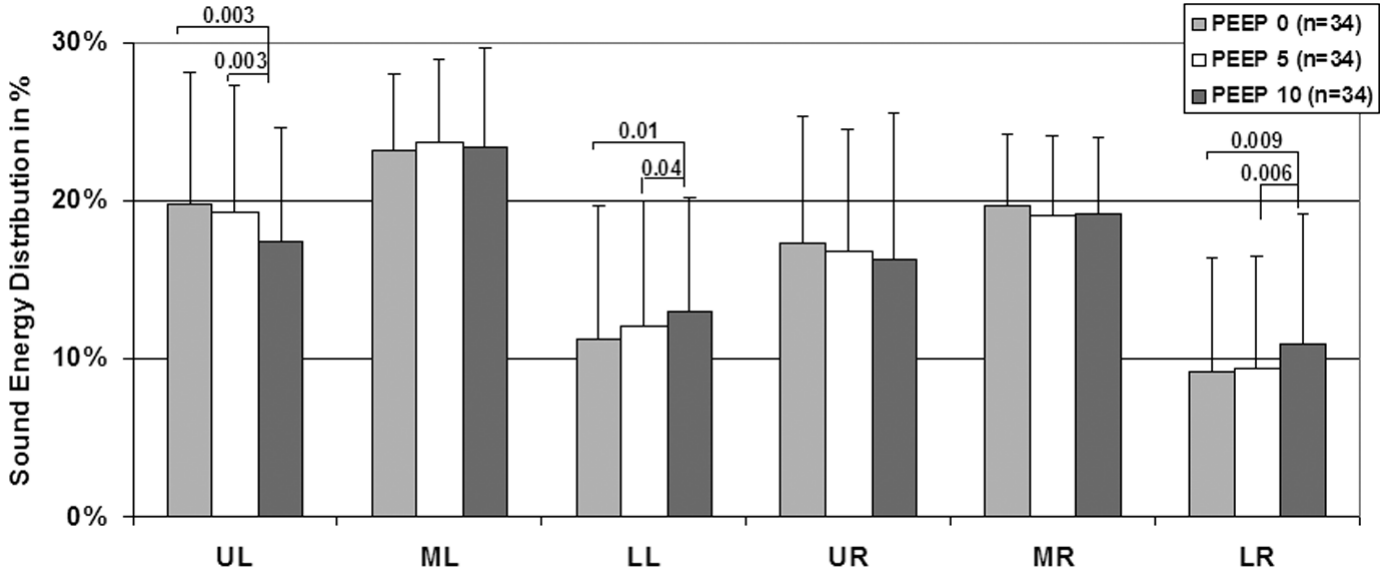
Mean ± standard deviation of sound energy distribution in 34 mechanically-ventilated patients recorded at three levels of PEEP (0, 5 and 10 cmH_2_O).  Significant *P *values are indicated (paired t-tests). LL = lower left; LR = lower right; ML = middle left; MR = middle right; PEEP = positive end-expiratory pressure; UL = upper left; UR = upper right.

Paired analysis conducted on the 34 patients for which recordings at PEEP 0, 5, and 10 cmH_2_O were available revealed that the proportion of sound energy in the diaphragmatic lung regions (LR and LL) was significantly increased with PEEP (*P *< 0.05, paired t-test), while the proportion of sound energy in the apical lung regions (UR and UL) was decreased (*P *< 0.05 in UL, paired t-test). The proportion of energy in the middle areas of the lungs (MR and ML) did not significantly change with PEEP (Figure 3). No additional shift was detected at PEEP 15 cmH_2_O (n = 15, Wilcoxon matched paired test). In patients with unilateral lung pathology (n = 13), the increase in sound energy in the diaphragmatic lung regions was significant in the diseased lung (7 ± 6% at PEEP 0 cmH_2_O versus 10 ± 7% at PEEP 10 cmH_2_O, *P *= 0.01, Wilcoxon matched-pairs) but not significant in the other lung (14 ± 8% at PEEP 0 cmH_2_O versus 15 ± 9% at PEEP 10 cmH_2_O, Wilcoxon matched-pairs). In patients with bilateral lung pathology (n = 21), the increase was significant in both lungs (*P *= 0.04).

The majority of the patients were ventilated on PSV or SIMV, spontaneous diaphragmatic activity was present in most of the patients. In order to assess the extent of this confounding factor, analysis was conducted according to the level of ventilator support provided to the patients. Patients were divided into two subsets according to the level of ventilator support needed (PSV < 15 cmH_2_O and PSV > 15 cmH_2_O). Sound energy distribution was compared between the two groups at each level of PEEP. No significant difference was detected (Wilcoxon two sample tests).

**Figure 4:**
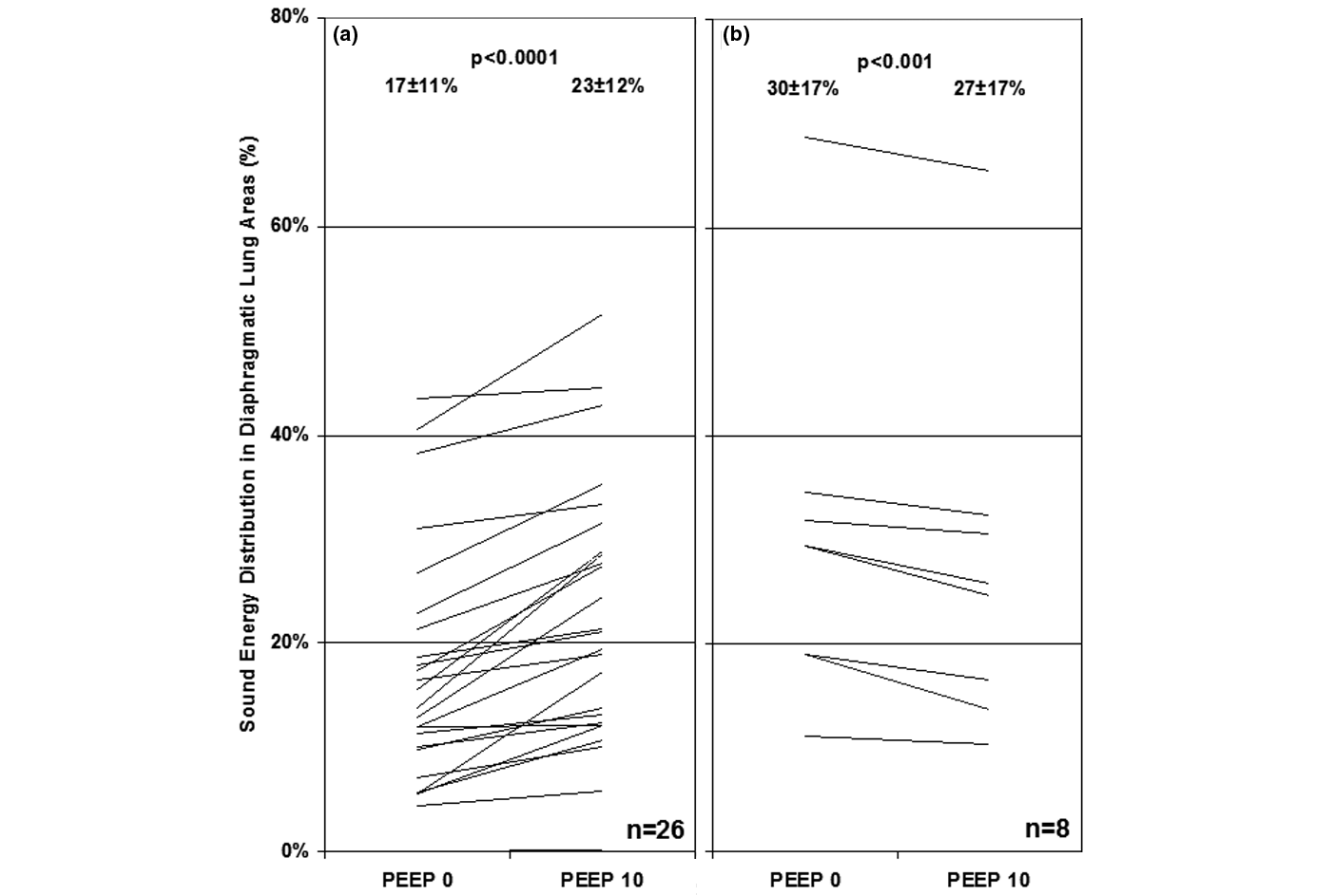
Individual sound energy distribution in diaphragmatic lung areas in 34 mechanically-ventilated patients recorded at PEEP levels 0 and 10 cmH_2_O.  Sound energy distribution increased from 17 ± 11% to 23 ± 12% (*P *< 0.0001) in **(a) **26 'responder' patients and decreased from 30 ± 17% to 27 ± 17% (*P *< 0.001) in **(b) **eight 'non-responder' patients. PEEP = positive end-expiratory pressure.

**Figure 5:**
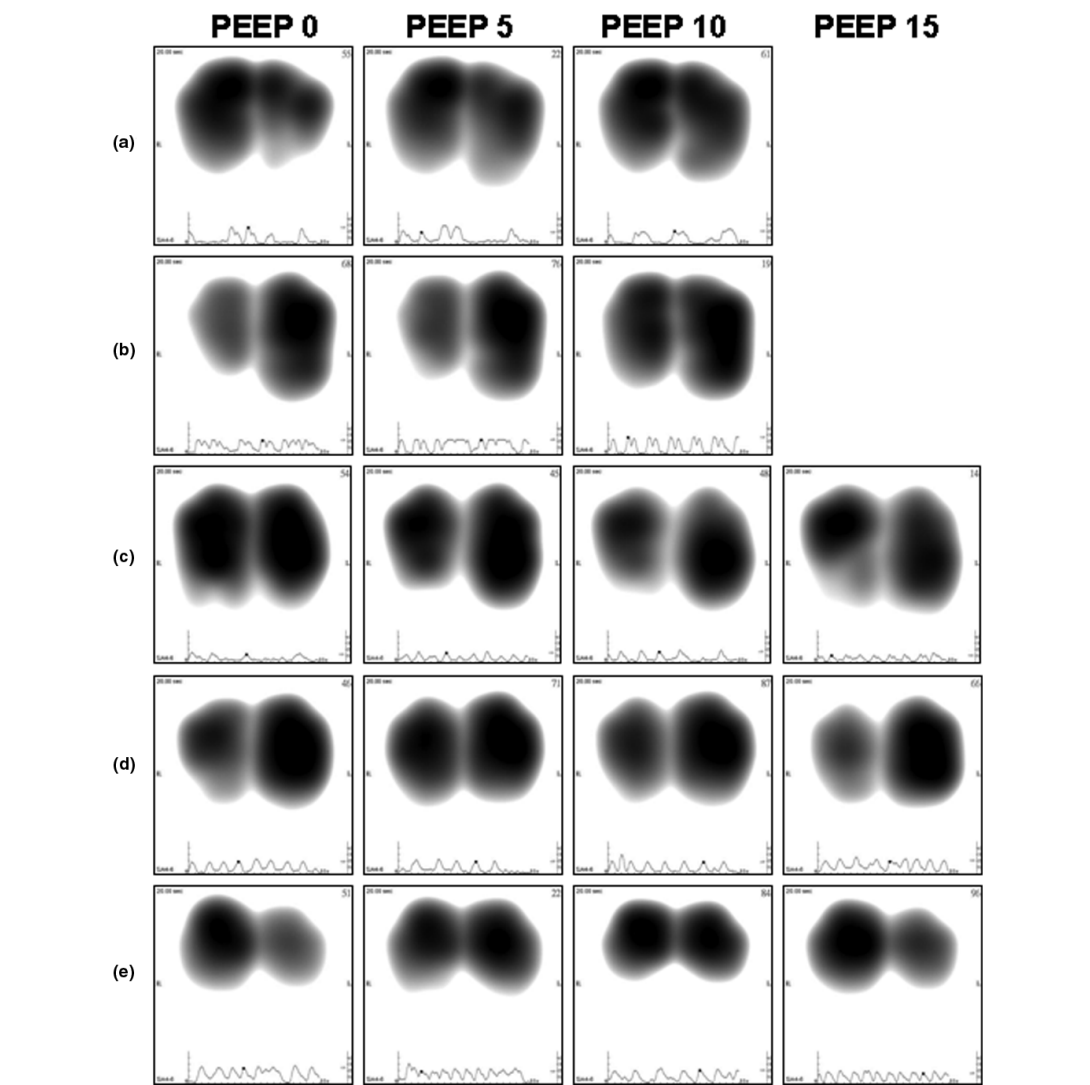
Representative frames (or maps) at peak-inspiratory flow obtained from five individual patients at PEEP levels 0, 5, 10 and 15 cmH_2_O.  **(a) **A 74-year-old female with respiratory failure. **(b) **A 19-year-old male with right pneumothorax. **(c) **A 83-year-old male with sternal wound infection. **(d) **A 77-year-old male with myasthenia gravis. **(e) **A 57-year-old male with acute pancreatitis. PEEP = positive end-expiratory pressure.

As shown in Figure 4, per patient analysis revealed that when increasing PEEP from 0 to 10 cmH_2_O, sound energy distribution increased in the diaphragmatic lung areas in 76% of the patients (26 of 34). In these cases, a larger peak-inspiratory flow image was obtained at higher PEEP (examples in Figure 5a and 5b). In several patients, an asymmetrical change of lung sound energy distribution was recorded at PEEP 15 cmH_2_O (Figure 5c, d, and 5e). Comparisons between VT, SpO_2_, Cdyn and Raw at two different levels of ADR are summarized in Table 2. When adjusted for RR, no difference in VT, Raw and SpO_2_was encountered between the two levels of ADR. At RRs lower than 20 breaths/minute, Cdyn tended to be higher for recordings with increased energy in the lower lung regions (ADR < 2). This difference approached significance (*P *= 0.058).

**Table 2:** Comparison between tidal volume, oxygen saturation, dynamic compliance, and airway resistance at two different levels of apico-diaphragmatic ratio

	**RR < 20 breaths/minute (15 ± 3)**			**RR > 20 breaths/minute (26 ± 5)**		
	**ADR < 2** **(n = 23)**	**ADR > 2** **(n = 14)**	***P *value**	**ADR < 2** **(n = 17)**	**ADR > 2** **(n = 23)**	***P *value**

**Tidal volume** **(ml)**	577 ± 91	528 ± 101	NS	479 ± 109	483 ± 135	NS
**Oxygen saturation** **(%)**	97 ± 3	97 ± 3	NS	97 ± 3	98 ± 2	NS
**Compliance** **(mL/mbar)**	60 ± 25	42 ± 12	0.058	51 ± 25	48 ± 20	NS
**Resistance** **(mbar L/second)**	15 ± 4	18 ± 7	NS	15 ± 3	16 ± 5	NS

Apico-diaphragmatic ratio (ADR) was defined as the ratio between the lung sound distribution in the apical lung areas (upper right (UR) + upper left (UL)) and the diaphragmatic lung areas (lower right (LR) + lower left (LL)) (ADR = (UL + UR)/(LL + LR)). Distribution was considered more heterogeneous if difference was larger than two, threshold derived from experience with healthy patients in supine position. *P *values are indicated (Wilcoxon two sample test) as well as non-significant (NS) data. RR = respiratory rate.

The repeatability of the measurement was assessed in 82 sets of double recordings obtained from 26 patients (20 double recordings at PEEP 0 cmH_2_O; 25 at PEEP 5 cmH_2_O; 26 at PEEP 10 cmH_2_O, and 11 at PEEP 15 cmH_2_O to a total of 164 recordings). Repeatability was performed by comparing the distribution of sound energy in each of the six lung regions of two repeated measurements, as well as in total left and right lungs. No significant difference was encountered between repeated measurements (paired t-test). Mean R^2 ^obtained for the different lung regions was 0.93 ± 0.02 (range 0.91 to 0.95) with a CV equal to 1.7%.

## Discussion

In this study, we used an acoustic-based monitoring system in order to assess possible shift in thoracic sound distribution during PEEP changes and to evaluate the repeatability of lung sound measurements in mechanically ventilated patients. Our results revealed a significant increase in sound distribution from the apical to the diaphragmatic lung areas when increasing PEEP from 0 to 10 cmH_2_O. This shift was especially pronounced in patients with severe lung pathology but was not affected by the level of pressure support needed. These statistical results were further supported by the analysis of the effect of PEEP on lung sound distribution in individual patients. As revealed in Figure 4, lung sound increased in the diaphragmatic lung areas in 76% of the patients.

The explanation for this acoustic phenomenon might be related to an increase in ventilation distribution in the diaphragmatic part of the lungs at higher levels of PEEP or to the effect of other PEEP-related physiologic factors, such as translocation of fluid from alveolar to interstitial spaces. A similar shift of lung sound distribution towards the base was recently described by Dellinger and colleagues [28], while changing mode of mechanical ventilation from volume control to pressure control and pressure support. The authors speculated that this shift was produced by a diaphragm-generated negative intrapleural pressure in pressure-targeted modes. The authors also proposed that the initial higher flow in pressure-targeted modes may serve to prime the proximal airway, allowing more time for slower, more laminar flow to produce a more homogenous distribution of air to lower lung regions. Correlation between lung condition and heterogeneity of lung sound distribution has been described in several additional studies. Bentur and colleagues [37] identified greater heterogeneity of lung sound distribution in pediatric patients with confirmed cases of foreign body aspiration when compared with healthy subjects. Lung sound heterogeneity was also described by Jean and colleagues [38] when comparing measurements performed on patients with normal lungs versus one diseased lung on chest radiograph. In agreement with the image normalization method used to generate the lung acoustic map, the authors explained that a larger image was considered to indicate a more homogeneous sound distribution and a smaller image a more focal distribution. In our study, the shift of lung sound distribution towards the base at PEEP 10 cmH_2_O was accompanied by an increase in the size of the peak-inspiratory flow image, in line with increased homogeneity of lung sound distribution. At PEEP level of 15 cmH_2_O, however, the lack of increased shift towards the base was adjunctive with a decrease in the size of the image as exemplified in Figures 5c to 5e. In light of the effect of PEEP elevation in lung sound distribution, comparison between measurements should be preferably performed when similar PEEP levels are applied.

**Figure 6:**
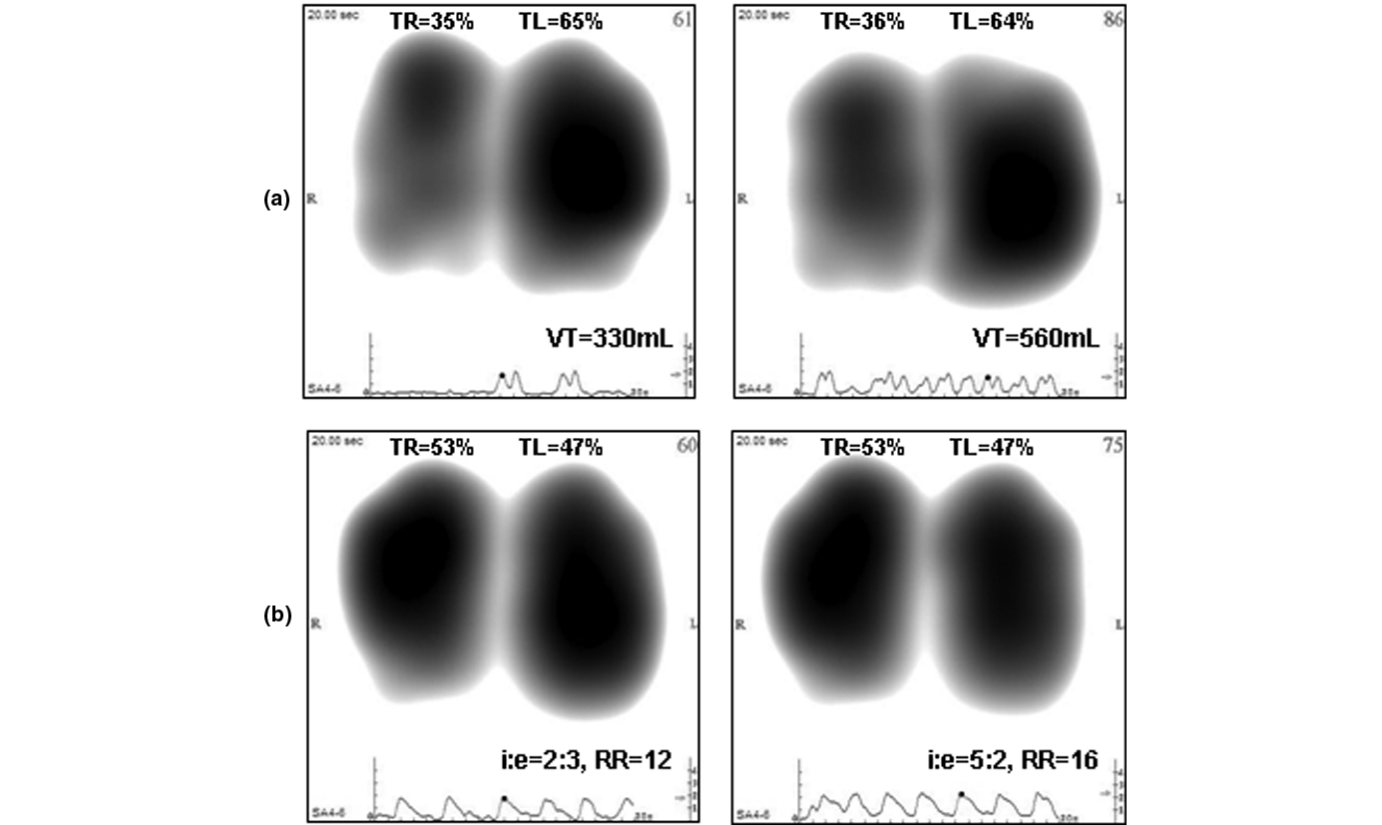
Representative frames (or maps) at peak-inspiratory flow obtained from two patients ventilated with different ventilator settings.  **(a) **A 72-year-old female with chronic obstructive pulmonary disease recorded at positive end-expiratory pressure (PEEP) level of 5 cmH_2_O and at two levels of tidal volume (VT; left = 330 mL, right = 560 mL); **(b) **A 24-year-old male with bilateral chest contusion recorded at PEEP level of 7 cmH_2_O, VT of 600 mL and at two levels of respiratory rate (RR) and inspiratory/expiratory ratio (i:e; left: i:e = 2:3 and RR = 12 breaths/minute, right: i:e = 5:2 and RR = 16 breaths/minute). TL = total left lung; TR = total right lung.

Repeatability of the lung acoustic measurements was comparable with that reported in healthy subjects [8,39,40]. This result in patients mechanically ventilated in pressure support mode may be *a priori *unexpected, especially when considering the variability of VT anticipated in this mode. However, this finding confirms that normalization of the acoustic distribution map reduces the mere effect of changes in ventilator settings when these changes do not affect the relative airflow distribution. Figure 6a, representing normalized images recorded from the same patient ventilated with two different VT, and Figure 6b, representing normalized images recorded from the same patient ventilated with two different airflow rates, further illustrate this finding.

The scope of this study was limited because of a restrictive protocol. Enrollment of deeply sedated patients mechanically ventilated in volume-controlled mode of mechanical ventilation may have allowed controlling for VT and inspiratory flow but this was out of the scope of this protocol. Another protocol-related limitation included the effect of volume history which may interfere with VT distribution. Moreover, differentiation between diaphragmatic redistribution induced by PEEP and VT-induced recruitment may be difficult. The population was heterogeneous and further studies should be performed on a more homogeneous population allocated to specific lung disease categories with emphasis on ALI or ARDS. Moreover, the heterogeneity of the clinical conditions exhibited by the patients at the time of investigation may be a limitation of the present study. Despite normalization, airflow velocity of the ventilators may have affected the results and, considering its impact on VT distribution and dynamic hyperinflation, it would have been interesting to consider the peak flow values. Furthermore, the protocol did not include a comparison of lung sound distribution with more appropriate tools, such as computerized tomography, functional residual capacity, or electrical impedance tomography. This should be investigated in the future. Although sometimes useful in research and clinical practice [41,42], the reliability of Cdyn is debatable, especially in non-paralyzed patients with non-uniform volume histories. Despite the fact that during pressure support mode of mechanical ventilation, Cdyn is particularly difficult to interpret, this parameter was used in this study because it was readily accessible in the scope of the protocol. In order to improve the accuracy of the measurement, three values were averaged at each time point. Finally, sound filtering to a band-pass of 150 to 250 Hz may have reduced the information as lung sound characteristics are contained in other frequency bands, especially above 250 Hz. Notwithstanding these limitations, the development of adjunctive technologies that assist in assessment of clinical benefits of PEEP and recruitment maneuver is still highly desirable [43,44].

## Conclusions

A shift in lung sound distribution from the apical to the diaphragmatic lung areas was observed during PEEP increase. This shift was not correlated with significant change in VT but was associated with an increase in Cdyn. High repeatability was obtained in this population. Further studies are needed in order to elucidate the mechanism of sound shift in relation to PEEP increment and to fully appreciate the contribution of PEEP increase to diaphragmatic sound redistribution.

## Key messages

• Sound distribution shifts from the apical to the diaphragmatic lung areas when increasing level of PEEP from 0 to 10 cmH_2_O.

• Acoustic shift is not correlated with significant change in VT but is associated with increased Cdyn.

• Sound distribution measurements are highly repeatable in mechanically ventilated patients.

## Abbreviations

ADR: apico-diaphragmatic ratio; ALI: acute lung injury; ARDS: acute respiratory distress syndrome; Cdyn: dynamic compliance; CV: coefficients of variation; FiO_2_: fraction of inspired oxygen; ICU: intensive care unit; LL: lower left; LR: lower right; ML: middle left; MR: middle right; PaO_2_: partial arterial pressure of oxygen; PEEP: positive end-expiratory pressure; PSV: pressure support ventilation mode; R^2^: coefficients of determination; Raw: airway resistance; RR: respiratory rate; SpO_2_: oxygen saturation; SIMV: synchronized intermittent mandatory ventilation; TL: total left lung; TR: total right lung; UL: upper left; UR: upper right; VRI: vibration response imaging; VT: tidal volume.

## Competing interests

Research materials for the VRI research program at Rabin Medical Center (Petah Tikva, Israel) are funded partially by Deep Breeze Ltd. SL has consultant agreement that includes honoraria and stock options (no current monetary value) with Deep Breeze Ltd and he was sponsored by GE Healthcare, Deep Breeze's distributor worldwide, to give lectures in academic meetings. YAG is an employee of Deep Breeze Ltd. IK, DD, JC, MG, MS, and PS declare that they have no competing interests.

## Authors' contributions

SL, YAG, IK, DD, MG, and MS participated in the design and coordination of the study and carried out the VRI recordings. SL and YAG worked on the data analysis. SL, YAG, JC, and PS drafted the manuscript. All authors edited and approved the final manuscript.
